# The second sodium pump: from the function to the gene

**DOI:** 10.1007/s00424-012-1101-3

**Published:** 2012-04-28

**Authors:** Miguel A. Rocafull, Luz E. Thomas, Jesús R. del Castillo

**Affiliations:** Laboratorio de Fisiología Molecular, Centro de Biofísica y Bioquímica, Instituto Venezolano de Investigaciones Científicas (IVIC), Apartado 20632, Caracas, 1020A Venezuela

**Keywords:** Ouabain-insensitive Na^+^-ATPase, ATNA, Furosemide, Hypertension, Inflammation, Na^+^ transport

## Abstract

Transepithelial Na^+^ transport is mediated by passive Na^+^ entry across the luminal membrane and exit through the basolateral membrane by two active mechanisms: the Na^+^/K^+^ pump and the second sodium pump. These processes are associated with the ouabain-sensitive Na^+^/K^+^-ATPase and the ouabain-insensitive, furosemide-inhibitable Na^+^-ATPase, respectively. Over the last 40 years, the second sodium pump has not been successfully associated with any particular membrane protein. Recently, however, purification and cloning of intestinal α-subunit of the Na^+^-ATPase from guinea pig allowed us to define it as a unique biochemical and molecular entity. The Na^+^- and Na^+^/K^+^-ATPase genes are at the same locus, *atp1a1*, but have independent promoters and some different exons. Herein, we spotlight the functional characteristics of the second sodium pump, and the associated Na^+^-ATPase, in the context of its role in transepithelial transport and its response to a variety of physiological and pathophysiological conditions. Identification of the Na^+^-ATPase gene (*atna*) allowed us, using a bioinformatics approach, to explore the tertiary structure of the protein in relation to other P-type ATPases and to predict regulatory sites in the promoter region. Potential regulatory sites linked to inflammation and cellular stress were identified in the *atna* gene. In addition, a human *atna* ortholog was recognized. Finally, experimental data obtained using spontaneously hypertensive rats suggest that the Na^+^-ATPase could play a role in the pathogenesis of essential hypertension. Thus, the participation of the second sodium pump in transepithelial Na^+^ transport and cellular Na^+^ homeostasis leads us to reconsider its role in health and disease.

## Introduction

The composition of body fluids depends on what is absorbed from the intestine and what is excreted in the sweat, breath, feces, and urine. The quantities and concentrations of electrolytes are mainly regulated by the kidney, since the intestine absorbs the vast majority of the water and salt that enter the intestinal lumen with no perceptible regulation.

Approximately 10 l of fluid pass through the intestine each day: 1.5–2.5 l is ingested, 1–1.5 l enters as saliva, 2–3 l is secreted in the stomach, and 1–2 l is secreted by the small intestine. Only 0.2 l is eliminated in the feces; the rest is reabsorbed in the small intestine and colon [93, 163]. The comportment of the intestine differs from one region to another. Thus, osmotic equilibrium is achieved in the proximal small intestine; net secretion occurs when the lumen of the proximal intestine contains a hypertonic fluid, whereas net absorption results when the content is hypotonic [56, 57]. There is net absorption of fluid and electrolytes from the resulting isotonic solution in the ileum and colon: The ileum absorbs approximately 9 l of isotonic fluid, whereas the colon absorbs 1 l of hypertonic solution. There is a distinct gradient of salt and water transport along the intestine. The unidirectional fluxes of salt and water in both directions are high in the duodenum and progressively diminish in the caudal direction. The fact that the absorptive flux decreases to a lesser extent than the secretory flux results in net absorption in the ileum and colon [175].

## Epithelial Na^+^ transport

Under optimal conditions for transport, the proximal sections of the intestine absorb salt and water more rapidly than the distal segments, when expressed per unit length of intestine but not per unit mucosal surface. In addition, the pores across which diffusion takes place are probably larger in the proximal than in the distal region of the intestine (approximately 7.5 Å in the jejunum and 3.5 Å in the ileum). This feature restricts the passive movement of solutes in the distal gut so they exert greater osmotic pressure [93, 163].

The movement of ions and water from the intestinal lumen to the blood along the paracellular pathway occurs principally by passive diffusion as a result of electrochemical gradients and the Starling forces inherent in the vascular network. As far as the coupled movement of water and sodium is concerned, it has been proposed that water movement is passive and responds to the osmotic gradient created by the active transport of salt by the cells [48, 72, 88, 185].

In “leaky” epithelia (e.g., small intestine and proximal tubule) with high water permeability, the relationship between the absorption of sodium and water is such that the fluid absorbed is always isotonic sodium, and water can pass from the lumen to the blood by two different pathways, i.e., paracellular and transcellular. In this respect, the small intestine is classed as a “leaky” epithelium, characterized by a relatively small transepithelial electrical potential difference, very low electrical resistance and high permeability to small ions and water. This ensures that the fluids secreted and absorbed are isotonic. The passive permeability of the epithelium is, in fact, determined by the tight junctions.

### Paracellular pathway

The paracellular pathway of the small intestine is extremely leaky to small ions, being only slightly selective for ions such as potassium. For instance, the permeability to K^+^ is about twice that to chloride, though the mobilities of these two ions in free solution are almost identical. In addition, there is relatively little discrimination between alkali metal ions. The relative permeabilities for Cs^+^/Rb^+^/K^+^/Na^+^/Cl^−^, determined in rabbit ileum, are 1.4:1.4:1.1:1.0:0.6. Moreover, the paracellular pathway is permeable to small molecules, such urea, arbinose, and xylose [82], and therefore, it behaves like an aqueous channel with a radius of 4.8 Å [50, 88, 149, 150, 152–154, 156, 175].

### Transcellular pathway

Sodium enters the enterocyte across the apical pole of the cell and is then pumped into the lateral spaces by active processes located in the basolateral plasma membrane. The increased local osmotic pressure in the intercellular space causes water to leave the cell and also probably to pass from the lumen, across the tight junction, directly into the lateral spaces. The osmotic pressure is thereby reduced, but the hydrostatic pressure is increased, resulting in a movement of solvent towards the capillaries because of the high hydraulic conductance of these spaces. This process is aided by the colloid osmotic pressure of the plasma proteins, which contributes to the forces moving water into the capillaries. This flow of water also causes the phenomenon known as “solvent drag,” in which solutes are moved across the small intestine as a result of the interaction of small molecules with the fluid stream moving across the intercellular space [73, 82, 85, 86].

The transcellular movement of sodium is known to be dependent on cellular energy and to involve the participation of carriers. The sodium ion enters the enterocyte in at least three ways. First, there is an electrogenic movement of sodium ions across the apical pole of the cell with no direct coupling to the movement of other solutes; in this case, the sodium movement is associated with passive absorption of chloride. Second, the entry is coupled with that of a wide variety of non-electrolytes. Thirdly, there is a coupled movement of Na^+^ and Cl^−^ across the brush-border membrane. Finally, sodium ions are actively extruded from the epithelial cells across the basolateral membrane against the electrochemical gradient.

#### Na^+^ entry to the intestinal epithelial cell

##### The electrogenic absorption of sodium

The cytoplasm maintains an electrical potential that is approximately 40 mV negative with respect to the solution bathing the mucosal face of the cell, and the intracellular sodium concentration is roughly one tenth of that in the mucosal and serosal fluids [5, 6, 81, 110, 152]. Sodium therefore enters the cell down an electrochemical gradient through sodium channels [60, 142, 146]. It is then expelled actively across the basolateral plasma membrane, as will be discussed in greater detail below. The active absorption of sodium is responsible for the maintenance of a small but significant transcellular potential difference of 3–5 mV (serosa positive), which drives the diffusional flux of chloride from mucosal to serosal fluid, either across the tight junctions or possibly also across the cell. In the mammalian intestine, sodium entry through Na^+^ channels is restricted to the colon [142, 146].

##### The coupled movement of sodium and organic solutes

The transport of a large range of water-soluble organic solutes, such hexoses, amino acids, vitamins, and bile salts, as well as diglycerides and triglycerides, depends on and is coupled to the absorption of sodium [14, 51, 59, 66, 147, 148, 152, 181, 182]. A ternary complex is formed in the brush-border membrane among a carrier, the substrate, and sodium ions, and this then crosses the membrane towards the interior of the cell as a result of the electrochemical gradient for sodium. Thus, the sodium gradient provides the energy necessary for the “uphill” transport of the solutes. These mechanisms were inferred from experiments with intact small intestine in vitro [151] and demonstrated unequivocally with the aid of brush-border membrane vesicles [75, 104, 144]. The sodium liberated into the cytoplasm is then pumped out of the cell actively across the basolateral plasma membrane, thus maintaining the low intracellular sodium concentration necessary for this type of transport on the one hand and the electrical force necessary for such a rheogenic process on the other [147]. The solute crosses the basolateral membrane by several mediated-transport mechanisms [14, 106, 183] from a region of higher concentration (the cytoplasm) to one of lower concentration (the basolateral space).

##### Neutral absorption of NaCl

Most of the sodium and chloride transport across the enterocyte entails an electroneutral process in which one sodium and one chloride ion are transferred in a coupled fashion. It was then suggested that the downhill movement of sodium would provide the energy necessary for the uphill transport of chloride ions, by direct analogy with the sodium-coupled transport of non-electrolytes. The chloride ion would leave the cell down an electrochemical gradient. An alternative model has been developed on the basis of experiments in vivo [170], brush-border membrane vesicles prepared from rat small intestine [29, 84, 105], and molecular characterization of the mechanisms [74, 94]. It has been suggested that the net NaCl absorption results from two processes in the apical membranes of the epithelial cells, exchange of Na^+^ and H^+^ and exchange of Cl^−^ and HCO_3_^−^. The bicarbonate and hydrogen ions are formed intracellularly from H_2_CO_3_ generated by the action of carbonic anhydrase, which is inhibited by acetazolamide. The downhill movement of sodium leads to a loss of H^+^ ions and therefore to an excess of base in the cytoplasm, which in turn leads to the downhill movement of bicarbonate in an outward direction and causes chloride to be accumulated, apparently against its electrochemical gradient. For this model to be valid, water and CO_2_ must be in thermodynamic equilibrium across the brush-border membrane. The two exchange systems must be interrelated and controlled by the intracellular pH. It is noteworthy that despite considerable efforts to locate a co-transport system for Na^+^ and Cl^−^ in brush-border membrane vesicles of small intestine and proximal tubule, evidence for such as system has only been found in the dogfish rectal gland [55], the urinary bladder of the teleost winter flounder [135], and the distal convoluted tubule of the mammalian kidney [58].

#### Na^+^ extrusion across the basolateral plasma membrane of epithelial cells

Sodium ions are pumped out of the epithelial cells across the basolateral membrane against their electrochemical gradient by a process that requires energy. It has been demonstrated that this energy is derived from the hydrolysis of ATP and that at least one enzyme is responsible for such hydrolysis: the ubiquitous Na^+^/K^+^-ATPase, which has been identified in all animal cells. Numerous experiments are consistent with this notion. The cardiac glycoside ouabain only inhibits the active absorption of sodium when added to the serosal face of the tissue [147]. The inhibition of transepithelial sodium transport is accompanied by a loss in cell potassium and a gain in sodium. In addition, autoradiographic [49, 161], histochemical [52], immunohistochemical [1, 65, 100], and cell fraction studies [75, 129] have localized the binding of ouabain and the activity of the Na^+^/K^+^-ATPase almost exclusively to the basolateral cell membrane, with little or no activity in the apical pole of the epithelial cell. However, there is evidence that the intracellular Na^+^ concentration and water content are not tightly linked to the function of the Na^+^/K^+^ pump. Studies of uni- or bilateral exposure of rabbit ileal mucosa to a K^+^-free solution on the intracellular concentrations of cations and cellular water have provided the following results [108]: (a) removal of potassium from the mucosal surface has no effect; (b) bilateral removal of potassium causes a reduction in intracellular potassium and an equivalent gain in intracellular sodium, with no change in cell water; and (c) in contrast, removal of potassium from the serosal medium leads to a reduction in cell potassium without concomitant changes in sodium and or water contents. These observations suggest that the maintenance of the high intracellular potassium and low intracellular sodium concentrations depend on the presence of potassium at the serosal face of the cell and that the apical cell membrane is impermeable to potassium ions. The removal of sodium ions from the mucosal or serosal solutions leads to a fall in intracellular sodium levels but affects neither the intracellular potassium concentration nor the flux of potassium across the basolateral membrane; the bilateral removal of sodium causes a reduction in both intracellular sodium and potassium, a decrease in cell water and a diminution of potassium movement across the serosal membrane. In addition, ouabain reduces cell potassium and increases cell sodium by equivalent amounts without changing the cell water content. These various data support the hypothesis that the Na^+^/K^+^ exchange pump is responsible for maintaining the normal intracellular concentrations of sodium and potassium, but appear to indicate that the regulation of cell volume is independent of this process.

Additionally, there are several indications that the active transport of sodium across the intestinal epithelial cell is not uniquely dependent on a Na^+^/K^+^ exchange pump. Even when intracellular sodium is depleted and its transepithelial movement is abolished by removal of this cation from the mucosal face of the tissue, there is no change in either intracellular potassium concentration or cell water, and the trans-serosal flux of potassium is unaltered [108]. These observations must mean that the fluxes of sodium and potassium are not closely coupled and that neither trans-epithelial sodium transport nor the regulation of cell water is entirely dependent on the Na^+^/K^+^ exchange pump.

In addition, solutes such as d-glucose and l-alanine strongly enhance the transcellular movement of sodium by stimulating the entry of the cation across the apical pole of the cell [151]. However, these organic solutes do not influence the rate of exchange of ^42^K^+^ across the basolateral membrane [108]. These observations agree with the findings of Lee & Armstrong [81], who measured the intracellular activities of Na^+^ and K^+^ in bullfrog small intestine using cation-selective microelectrodes and observed that in the presence of 3-*O*-methyl-glucoside the ion activities were significantly reduced, despite the stimulation of transcellular sodium transport by this sugar. If there were an absolute relationship between the transport of sodium and the Na^+^/K^+^ exchange pump, an increase in cell potassium would be predicted. Indeed, these observations have been confirmed with isolated cells [155], where non-metabolizable hexoses elicited no rise in cell potassium. More recently, it has been proposed that changes in the rate of Na^+^ entry across the apical membrane, which should result in changes in the rate of basolateral membrane Na^+^/K^+^-pump activity and Na^+^ absorption, are accompanied by parallel changes in the K^+^ conductance across the basolateral membrane through K^+^ channels [68], avoiding the increase in intracellular potassium and hyperpolarizing the cell, which would induce Cl^−^ exit. This KCl extrusion would permit the cell volume to be regulated. However, this hypothesis does not explain volume regulation in the presence of serosal ouabain.

The small intestine is not the only epithelium where there appears to be no strict relationship between transcellular sodium transport and sodium–potassium exchange, and indeed, findings of this nature were made early by numerous authors [11, 19, 30, 53, 54, 62, 91, 92, 137–139].

These observations suggest the existence of a second transport mechanism, independent of the Na^+^/K^+^-pump, which actively extrudes sodium across the basolateral plasma membrane of intestinal and renal epithelia.

## Identification of a second sodium pump

In the proximal tubular cell of the guinea pig kidney, two different mechanisms for sodium transport across the basolateral membrane have been described and characterized [125, 126, 180]. One pump exchanges intracellular sodium for extracellular potassium, while the other actively expels sodium, passively followed by chloride ions and water. The former of these pumps is strongly inhibited by ouabain, weakly inhibited by ethacrynic acid and insensitive to furosemide and triflocin, whereas the second is refractory to ouabain but inhibited by ethacrynic acid, furosemide, and triflocin. Both processes are dependent on cellular energy since they are suppressed by 2,4-dinitrophenol or anoxia, indicating that they derive their energy from the hydrolysis of ATP. Similar mechanisms have been identified and characterized in isolated guinea pig small intestinal cells [46] and everted rat jejunum [167].

The enterocyte regulates its Na^+^ content by two pumps located in the basolateral plasma membrane. One exchanges Na^+^ for K^+^, is inhibited by ouabain, and insensitive to ethacrynic acid and furosemide. The second transports Na^+^ with Cl^−^ and water, is insensitive to ouabain, but is inhibited by ethacrynic acid and furosemide. These results confirmed the evidence from experiments with inside–out basolateral plasma membrane vesicles from guinea pig small intestinal epithelial cells [43], rat jejunum [166] and rat proximal tubule [96], where two distinct mechanisms capable of accumulating sodium in the intravesicular space were demonstrated when ATP was added to the incubation medium. One transports sodium actively in the absence of potassium, whereas the other requires potassium to be present within the vesicles. The two mechanisms can also be differentiated by their affinities for sodium, their optimal pH, and their behavior towards different inhibitors. Thus, the active mechanism that transports sodium in the absence of potassium is refractory to ouabain but is inhibited by ethacrynic acid and furosemide, while the mechanism that causes sodium accumulation in the vesicles in the presence of internal potassium is strongly inhibited by ouabain, weakly inhibited by ethacrynic acid, and insensitive to furosemide. ATP is a specific stimulator of both processes and the requirement for magnesium is absolute in both cases.

These two active Na^+^ transport mechanisms, identified in epithelial cells of the small intestine and proximal tubule, are associated with ATPase activities located in the basolateral plasma membranes of such cells. The two Mg^2+^-dependent, sodium-stimulated ATPase activities have been identified in microsomal fractions [116] and crude basolateral plasma membrane fractions of the renal proximal tubule [45, 117] and purified basolateral plasma membranes of small intestinal cells [44]. In these preparations, the Na^+^-ATPase is stimulated by sodium alone or to a lesser extent by Li^+^, whereas the Na^+^-K^+^-ATPase requires both sodium and potassium for activation. These facts link the enzymes to the sodium transport systems. The Na^+^-ATPase specifically hydrolyzes ATP, as does the Na^+^-K^+^-ATPase, though the latter has some effect on GTP and ITP. This property defines the two enzymes as ATPases. The fact that the enzyme is stimulated indifferently by different sodium salts essentially excludes the possibility that the Na^+^-ATPase is an anion-stimulated ATPase, whose existence has been postulated [61]. The Na^+^-ATPase and the Na^+^-K^+^-ATPase can also be differentiated by their slightly different pH optima and different sensitivities to pH. They also reveal somewhat different affinities for sodium, the apparent *K*_m_ values for sodium being 8–9 and 15–18 mM, respectively.

The two enzymes can also be distinguished by their different behavior towards a series of inhibitors. The Na^+^-ATPase is insensitive to ouabain but is inhibited by ethacrynic acid and furosemide and triflocin; in contrast, the Na^+^/K^+^-ATPase is fully inhibited by ouabain, partially inhibited by ethacrynic acid and unaffected by furosemide or triflocin. These features are of extreme importance, since they correspond exactly to the sensitivities of the two sodium-transporting mechanisms that have been characterized in renal [125, 180] and isolated small intestinal [46] cells. This correspondence provides the strongest evidence that each of the enzymes represents the machinery responsible for each one of the transport systems.

**Fig. 1:**
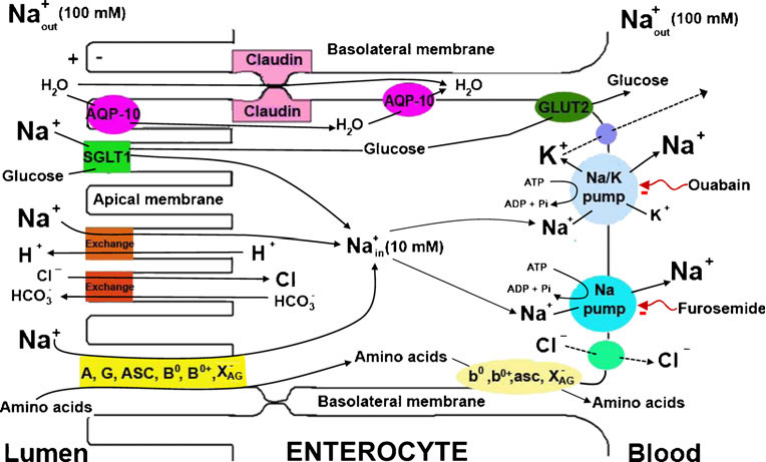
Theoretical model for transepithelial Na^+^-transport across the intestinal and renal epithelia. In the small intestine and renal proximal tubule, transepithelial Na^+^-transport depends on: (a) Na^+^ entry across the luminal membrane of the epithelial cells, following its electrochemical gradient and (b) Na^+^ extrusion through the basolateral plasma membrane by active mechanisms. The primary active Na^+^ transport has been mainly attributed to the Na^+^/K^+^ pump. However, two different mechanisms for active Na^+^ transport across the basolateral plasma membrane have been described in small intestinal and proximal tubular cells. One mechanism, corresponding to the Na^+^/K^+^ pump, requires K^+^, is inhibited by ouabain but is insensitive to furosemide. The other does not require K^+^, is insensitive to ouabain but inhibited by furosemide, and extrudes sodium with Cl^−^ and water. This K^+^-independent, ouabain-insensitive mechanism had been denominated the second sodium pump and has been implicated in isosmotic cell volume regulation. These Na^+^ pumps have been associated with two different ATPase activities identified in the basolateral plasma membranes of enterocytes and proximal tubular epithelial cells. The Na^+^/K^+^ pump is linked to the Na^+^/K^+^-ATPase, which is Mg^2+^-dependent, stimulated by Na^+^ and K^+^, sensitive to ouabain and vanadate but insensitive to furosemide; while the K^+^-independent Na^+^ pump has been associated with the Na^+^-ATPase, which is Mg^2+^ dependent, specifically stimulated by Na^+^, does not require K^+^, and is insensitive to ouabain but inhibited by furosemide. This basolateral active Na^+^-transport, coupled to the passive Na^+^ entry through apical co-transporters, energizes the absorption of osmotically active solutes followed by water. This process generates isosmotic absorption of sodium, solutes and water

A model has been developed to explain the transepithelial transport of Na^+^ across the intestine (Fig. 1).

## Identification of the ouabain-insensitive Na^+^-ATPase in different animal tissues

The ouabain-insensitive, Mg^2+^-dependent Na^+^-ATPase activity has also been identified in different animal tissues [102, 122]: arterial vascular muscle cells [115]; mammalian brain microsomal fractions [165]; sea bass (*Dicentrarchus labrax L.*) gills [12] and kidney [113]; squid gill microsomes [120]; shrimp (*Macrobrachium amazonicum*) gill homogenates [127]; gilthead bream (*Sparus auratus L*.) gills [173]; freshwater mussel (*Anodonta cygnea*) gills [77]; rainbow trout (*Oncorhynchus mykiss Walbaum*) gills [174]; rabbit cardiac sarcolemma [18]; malpighian tubules from *Rhodnius prolixus* [24, 25]; Trypanosoma cruzi epimastigotes [21, 145]; cultured MDCK I cells [39]; *Entamoeba histolytica* [38]; *Leshmania amazonensis* [36]; and pig kidney [79]. Recently, the Na^+^-ATPase activity has been reported in homogenates of several rat tissues [136].

The identification of an ouabain-insensitive Na^+^-ATPase in different animal species and tissues is very interesting because it suggests that the pump is universally distributed. However, the genes related to each of these enzymatic activities have to be characterized before the ubiquity of this ATPase can be accepted. For instance, the gene encoding the ouabain-insensitive Na^+^-ATPase in *T. cruzi* (TcENA or TrENA) [87] is different from that in mammals (*atna*) [140]. Alignment of *atna* and TcENA (by ClustalW) reveals that they encode different proteins. TcENA is much longer than ATNA. They only have 24 % identity, mainly related to the eight P-type ATPase motifs that they share. In addition, the binding site for the first cation has a significant modification. In fact, TcENA is a P-type ATPase more related to plant [158] or fungal [10] Na^+^-ATPases. Moreover, TcENA is functionally different from ATNA. TcENA is stimulated by Na^+^ and K^+^, while ATNA is specifically activated by Na^+^.

## Modulation of the Na^+^-ATPase activity

The activity of the ouabain-insensitive, Mg^2+^-dependent Na^+^-ATPase can be modulated by several physiological conditions. Among the most relevant are:

### Cell volume

Under isotonic conditions, there is a close relationship between the cell volume and the activity of the ouabain-insensitive Na^+^-pump, whereas the Na^+^/K^+^-pump activity is not affected by variations in cell volume [118]. The Na^+^-pump activity (Na^+^ transport and Na^+^-ATPase activity) is minimal when the cell water content is low but increases when the cell water content rises [124]. In addition, basolateral plasma membranes prepared from swollen proximal tubule cells of rat kidney show an ouabain-insensitive Na^+^-ATPase activity ten times higher than membranes isolated from control cells. If the swollen cells recover their volume, the activity decreases tenfold to control values.

### High NaCl diet

High dietary NaCl intake induced an increase in the activity of the ouabain-insensitive Na^+^-ATPase. Healthy male rats exposed to chronic ingestion of isotonic NaCl solution for 4 months presented an increase (about 70 %) in the activity of the ouabain-insensitive Na^+^ pump in the basolateral plasma membranes of the kidney proximal tubular cells, whereas the ouabain-sensitive Na^+^/K^+^-pump activity did not change [95]. In addition, the ouabain-insensitive Na^+^-ATPase activity of kidney proximal tubular cells from rats fed with a high-Na^+^ diet for 4 months increased, while the Na^+^/K^+^-ATPase was not altered [111]. Moreover, proximal tubular kidney cells from rats chronically fed for 15 months with isotonic NaCl solution showed increases in kidney volume and in Na^+^ and Cl^−^ content, as well as the activity of the ouabain-insensitive Na^+^-ATPase in the basolateral plasma membranes. These effects were reversed by returning the rats to drinking tap water. The authors propose that the Na^+^-ATPase activity is modulated in vivo by the cell volume [47].

### Aging

The active Na^+^ transport mediated by the Na^+^/K^+^-pump and the active Na^+^-extrusion with Cl^−^ and water through the second sodium pump were lower in old rats (24 months) than young ones (3 months). The oxygen consumption associated with each of the two active mechanisms of Na^+^ extrusion was also diminished in the old rats [123]. However, the turnover rate of the (Na^+^/K^+^)-ATPase was diminished by aging (about 40 %), while the Mg^2+^-dependent Na^+^-ATPase activity was similar in the kidneys of young and old rats, in both homogenates and basolateral plasma membrane fractions [97]. In contrast, it has been reported that the Na^+^- and Na^+^/K^+^-ATPases in jejunum epithelial cells have the same characteristics in the basolateral membrane of the enterocyte throughout the lifespan of the animal, but they quantitatively decrease with aging [168].

### Angiotensins

Angiotensin II (Ang II) stimulates the Na^+^-ATPase activity in outer kidney cortex kidney [130], mediated by AT1 receptors through the PI-PLCβ/PKC pathway [40, 131–133]. Additionally, it has been shown, in inner kidney cortex, that Ang II inhibits the Na^+^-ATPase activity, mediated by AT2 receptors through a cholera toxin-sensitive PKA pathway [37]. These receptors are differentially distributed throughout the nephron, from outer to inner renal cortex, leading to a preferential binding of Ang II either to AT1 or AT2 receptors, respectively. Therefore, the predominant effect of Ang II on the Na^+^-ATPase in outer cortex would be stimulatory [40, 131–133], while in the inner cortex this peptide would have an inhibitory effect [37].

Ang-(1–7), as has been indicated for Ang II, has a dual effect on the Na^+^-ATPase. It selectively stimulates the enzyme in basolateral membranes of renal proximal tubules through AT1 receptors [23]. Moreover, experiments in which the AT1 receptors were blocked by losartan (an inhibitor of AT1 receptors) showed that Ang-(1–7) inhibits the proximal tubule Na^+^-ATPase by its interaction with AT2 receptors, which subsequently activate the G_i/o_/cGMP/PKG pathway [35].

It is noteworthy that the stimulatory effect of Ang II in proximal tubule is reversed by Ang-(1–7) via Ang-(1–7)-specific receptors [78].

### Nucleosides

Adenosine and inosine are purine nucleosides that modulate several physiological processes. Cellular signaling by adenosine occurs through four known receptor subtypes (A1, A2A, A2B, and A3). In the proximal tubule, adenosine decreases the activity of the ouabain-insensitive Na^+^-ATPase interacting with A1 subtype receptors through G_i_ protein pathway, without effect on the Na^+^/K^+^-ATPase [22]. Furthermore, in the presence of A1 selective antagonist, adenosine stimulates the Na^+^-ATPase, effect mediated by A2A receptors through PKA pathway [179].

Although the activation of PKA- or PKC-signaling pathways separately stimulates the Na^+^-ATPase activity [131, 179], the PKA pathway seems to be involved in a negative modulation of PKC-stimulatory effect when both ways are sequentially activated [63, 64]. Thus, the stimulatory effect of Ang II, mediated by PKC pathway, is reversed by adenosine through PKA pathway [63, 64]. In consequence, the existence of both stimulatory and inhibitory PKA-mediated phosphorylation sites in the Na-ATPase has been proposed [64]. The phosphorylation of the Na^+^-ATPase by PKC may induce a conformational change in the protein, which on turn might lead to exposure of inhibitory PKA-targeted sites. The phosphorylation of these inhibitory sites by PKA would reverse the stimulatory effect induced by PKC [64].

Inosine inhibits the renal ouabain-insensitive Na^+^-ATPase, an effect mediated by A1 receptor via G_i_ protein pathway [7].

### Bradykinin

Bradykinin (BK), a peptide of nine amino acids, is a potent endothelium-dependent vasodilator that causes natriuresis. It has been reported that BK stimulates the ouabain-insensitive Na^+^-ATPase activity in kidney cortex homogenates (2.2 times) but inhibits the enzyme in basolateral membrane preparations by 60 %. The stimulation of the Na^+^-ATPase activity occurs through the interaction with B1 receptors, while the inhibitory effect on the enzyme is mediated through B2 receptors [27]. The effect of BK is mediated by activation of phosphoinositide-specific PLC β/PKC. The inhibitory effect is mediated by Ca^2+^-independent phospholipase A2, arachidonic acid (AA), and PGE2 [83], and seems to involve G-protein and PKA activation. Finally, it is interesting that BK counteracts the stimulatory effect of Ang-(1–7) on the proximal tubule Na^+^-ATPase activity through the B2 receptor [26, 89].

### Purine bases

Adenine [177] and guanine [178] decrease the activity of the renal ouabain-insensitive Na^+^-ATPase through Gi protein-coupled receptors.

### Urodilatin and atrial natriuretic peptide

Atrial natriuretic peptide (ANP) and urodilatin specifically inhibit the Na^+^-ATPase activity by activating the PKG pathway through the natriuretic peptide receptor (NPR-A) located in the luminal and basolateral membranes of proximal tubular cells [28, 176].

### Epinephrine

It has been shown that norepinephrine stimulates the furosemide-sensitive Na^+^ pump and partially inhibits the ouabain-sensitive Na^+^/K^+^ pump, apparently through intracellular Ca^2+^ increase [119]. These effects are associated with both α- and β-adrenergic receptors [184]. In this sense, it has been shown that Ca^2+^ in the micromolar range stimulates the Na^+^-ATPase and partly inhibits the Na^+^/K^+^-ATPase of basolateral plasma membranes from guinea pig kidney [121], as well as the furosemide-sensitive ATP-induced Na^+^ transport in basolateral plasma membrane vesicles of rat kidney cortex [98], suggesting that Ca^2+^ could regulate the magnitude of Na^+^ extrusion with Cl^−^ and water in proximal tubule epithelial cells.

### Leptin, nitric oxide, ROS, and cyclic nucleotides

Chronic hyperleptinemia, induced by repeated subcutaneous leptin injections, increased cortical Na^+^/K^+^-ATPase, medullar Na^+^/K^+^-ATPase, and cortical Na^+^-ATPase [8]. This effect was prevented by co-administration of the superoxide dismutase mimetic tempol or the NADPH oxidase inhibitor apocynin. Acutely administered NO• donors decreased the Na^+^-ATPase activity. This effect was abolished by the soluble guanylate cyclase inhibitor ODQ (1H-[1, 2, 4]oxadiazolo[4,3,-a]quinoxalin-1-one), but not by PKG inhibitors. Exogenous cGMP reduced Na^+^-ATPase activity, but its synthetic analogues, 8-bromo-cGMP and 8-pCPT-cGMP, were ineffective. The inhibitory effect of NO• donors and cGMP was abolished by an inhibitor of cGMP-stimulated phosphodiesterase. An exogenous cAMP analogue and dibutyryl-cAMP increased the Na^+^-ATPase activity and abolished the inhibitory effect of cGMP. Finally, the administration of a superoxide-generating mixture (xanthine oxidase plus hypoxanthine) increased the Na^+^-ATPase activity. These results suggest that nitric oxide decreases renal Na^+^-ATPase activity by stimulating cGMP, which in turn activates PDE2 and decreases the cAMP concentration. Increased production of reactive oxygen species may lead to the stimulation of Na^+^-ATPase activity by scavenging NO• and limiting its inhibitory effect. The authors suggest that chronic hyperleptinemia is associated with an increase in Na^+^-ATPase activity due to excessive oxidative stress [9].

### Lipid peroxidation and ethanol

It has been shown that lipid peroxidation [99] and ethanol [143] inhibit the Na^+^-ATPase.

### Ceramide

Ceramide-activated PKA and PKC zeta inhibit the Na^+^-ATPase of the kidney proximal tubule [17].

### Hypertension

The ouabain-insensitive Na^+^-ATPase activity and its regulation by Ang II in spontaneously hypertensive rats (SHR) has been evaluated [128]. Na^+^-ATPase activity was enhanced in 14-week-old but not 6-week-old SHR. The addition of Ang II decreased the enzyme activity in SHR to a level similar to that obtained in the Wistar–Kyoto rats used as controls. The inhibitory effect of Ang II was completely reversed by a specific antagonist of the AT2 receptor. Treatment of SHR with the AT1 receptor inhibitor losartan for 10 weeks (weeks 4–14) prevented the increase in Na^+^-ATPase activity observed in 14-week-old SHR. These results indicate a correlation between AT1-receptor activation and the increased ouabain-insensitive Na^+^-ATPase activity in SHR.

**Fig. 2:**
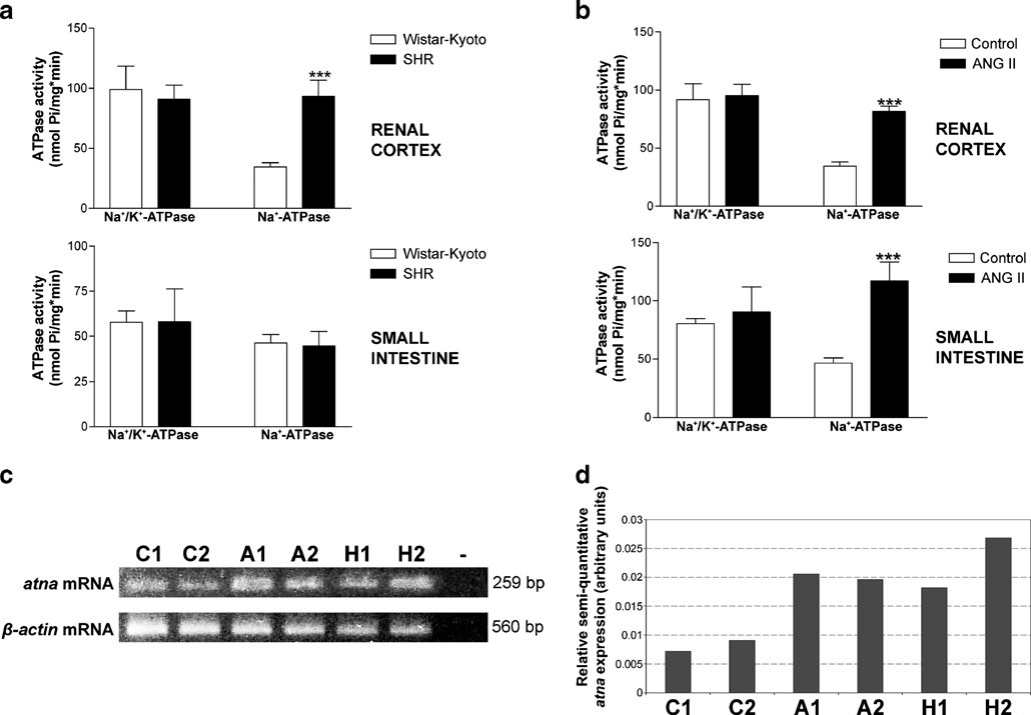
Regulation of the Na^+^-ATPase by angiotensin II and its possible role in arterial hypertension. **a** Na^+^- and Na^+^/K^+^-ATPase activities in renal cortex (*upper graphic*) and small intestine (*lower graphic*) from Wistar–Kyoto (control, *open columns*) or spontaneously hypertensive (SHR, *closed columns*) rats (200–250 g weight). Na^+^-ATPase activity is increased in the renal cortex of SHR. **b** Na^+^- and Na^+^/K^+^-ATPase activities in renal cortex (*upper graphic*) and small intestine (*lower graphic*) from Wistar–Kyoto rats treated (Ang II, *closed columns*) or not (control, *open columns*) with angiotensin II, administered through osmotic pumps at a dosage of 400 ng/kg body weight/day for 2 weeks. Na^+^-ATPase activity is increased in the renal cortex and small intestine of rats treated with angiotensin II. **c**, **d** Semi-quantitative expression level of Na^+^-ATPase mRNA (*atna*) in different rat specimens. Total RNA from untreated Wistar–Kyoto (control, specimens C1 and C2), Wistar–Kyoto treated with angiotensin II (specimens A1 and A2) and SHR was isolated using Trizol reagent and following the manufacturer’s indications. The first cDNA strand was synthesized through the Thermoscript RT-PCR system, using Oligo(dT)20 as primer. The PCR step was carried out with specific primers for Na^+^-ATPase or β-actin cDNA (house-keeping gene), as described [140]. Electrophoretic analysis of the PCR product on 2 % agarose gels (**c**) and the respective densitometric quantification (**d**) are shown. *Bars* represent the relative Na^+^-ATPase mRNA expression normalized to the β-actin house-keeping gene as arbitrary units. The Na^+^-ATPase mRNA is over-expressed in rats treated with angiotensin II and in SHR

Our group has obtained evidence indicating that the Na^+^-ATPase activity is increased in basolateral plasma membranes of renal cortex from spontaneous hypertensive rats but not in the small intestine (Fig. 2a). Systemic treatment with Ang II increased the Na^+^-ATPase activity in both renal and small intestinal tissues (Fig. 2b). In agreement, the *atna* gene is overexpressed in renal cortex from SHR and Ang II-treated rats (Fig. 2c, d). These data suggest that the Na^+^-ATPase could be important in the pathogenesis of essential hypertension.

The multiple modulation of the activity of the Na^+^-ATPase suggests the relevance of this enzyme to renal and intestinal sodium homeostasis.

## Isolation and characterization of the intestinal ouabain-insensitive Na^+^-ATPase

Despite the extensive biochemical, functional, and pharmacological evidence indicating the existence and the physiological relevance of the ouabain-sensitive Na^+^-ATPase in different tissues, no particular protein or gene related to ATPase activity had been identified until recently. Our group has been able to solubilize both the Na^+^- and Na^+^/K^+^-ATPases from the enterocyte basolateral plasma membrane without inactivation, to separate them physically using Con-A affinity chromatography and to purify the Na^+^-ATPase by anion-exchange chromatography [140]. The purified enzyme retains the functional characteristics of the native enzyme, e.g., Mg^2+^ dependence, specific stimulation by sodium, insensitivity to ouabain, and inhibition by furosemide and vanadate. Electrophoretic analysis and anion-exchange chromatography demonstrate that the Na^+^-ATPase is a protein complex comprising at least two subunits of 90 kDa (α-subunit) and 50 kDa (β-subunit). The 50 kDa subunit is glycosylated and could be a previously undescribed P-type ATPase β-subunit. Although the available sequence evidence is not conclusive, its N-terminal sequence (SPLEYQD) does not correspond to any previously reported β-subunit [140].

**Fig. 3:**
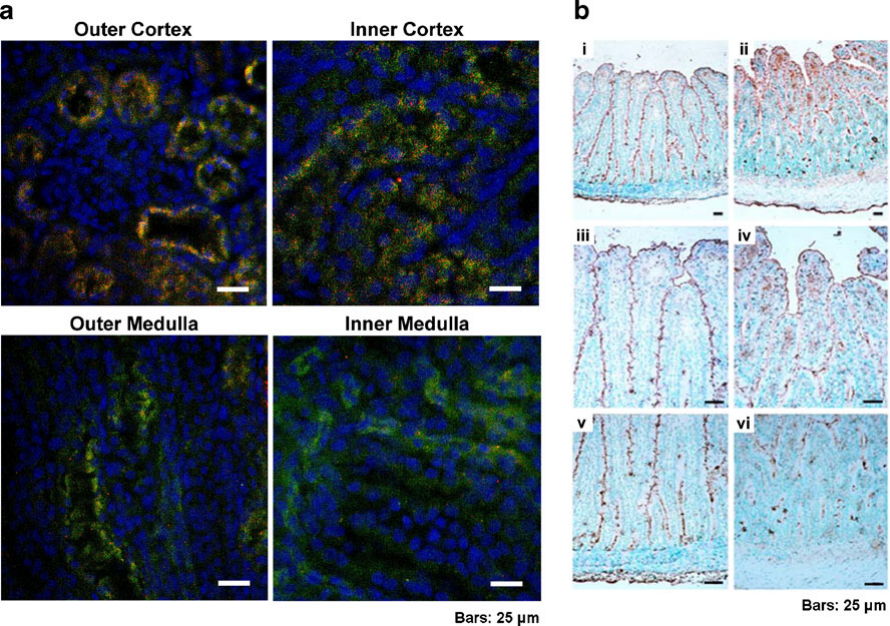
Immuno-localization of the Na^+^- and Na^+^/K^+^-ATPases in intestinal and renal tissues. Kidney and small intestine (jejunum) were obtained from guinea pig (*Cavia porcellus*), embedded in Cryomatrix, frozen in liquid nitrogen and sectioned (8 μm) in a cryostat microtome at −20°C and mounted on glass slides coated with poly-l-lysine. Sections were fixed in acetone at −20°C, dried in air, and treated for immune-fluorescence or immune-histochemistry. **a** Immunolocalization of Na^+^- and Na^+^/K^+^-ATPases in different kidney slices, as indicated (outer cortex and medulla, inner cortex, and medulla). Na^+^-ATPase was detected using its IgY polyclonal antibodies and a goat polyclonal anti-chicken IgG labeled with tetramethyl-rhodamine isothiocyanate (*red*), while Na^+^/K^+^-ATPase was identified using a horse anti-Na^+^/K^+^-ATPase polyclonal antibody and a rabbit polyclonal anti-horse IgG labeled with fluorescein isothiocyanate (*green*). **b** Immunohistochemistry of guinea pig jejunum using anti-Na^+^/K^+^-ATPase (**a**, **c**, **e**) or anti-Na^+^-ATPase (**b**, **d**, **f**) IgY polyclonal antibodies. Sections were incubated with a rabbit polyclonal anti-chicken-IgY conjugated with peroxidase (1:1,000 dilution in PBS). Note the differential distribution of Na^+^- and Na^+^/K^+^- ATPases in both renal and intestinal tissues

**Fig. 4:**
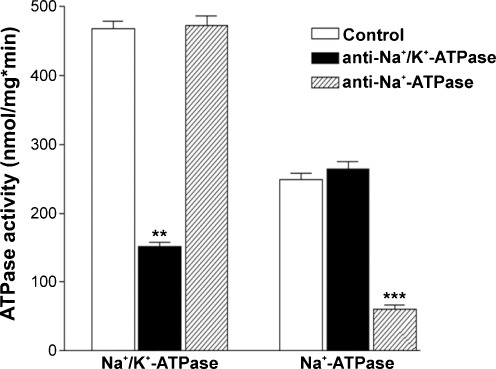
Effect of anti-Na^+^-ATPase and anti-Na^+^/K^+^-ATPase IgY polyclonal antibodies on sodium ATPase activities. Basolateral plasma membranes from small intestine, obtained as described [41], were pre-incubated with pre-immune IgY (control, *open bars*) or anti-Na^+^-ATPase IgY polyclonal antibodies (anti-Na^+^-ATPase, *ruled bars*) or anti-Na^+^/K^+^-ATPase IgY polyclonal antibodies (anti-Na^+^/K^+^-ATPase, *closed bars*) in a 1:500 dilution for 30 min at 4°C. Each antibody specifically inhibited the ATPase from which it was developed

As shown in Fig. 3, the distribution of the Na^+^- and Na^+^/K^+^-ATPase differs through distinct guinea pig kidney segments. Both enzymes are well expressed in the outer cortex, but Na^+^-ATPase expression is lower in the inner regions of the kidney and absent in the medulla. In the intestine, the Na^+^-ATPase is mainly expressed in villous (small intestine) and surface (colon) cells. In the crypt region (immature cells), the enzyme seems to have an intracellular distribution. This particular renal and intestinal distribution probably has to do with the physiological role of this enzyme in sodium transport in these epithelia. Furthermore, IgY polyclonal antibodies raised against the purified Na^+^- and Na^+^/K^+^-ATPases differentially recognize these enzymes. Antibodies raised against the purified Na^+^-ATPase inhibit the Mg^2+^-dependent ouabain-insensitive Na^+^-stimulated ATPase activity without effect on the Na^+^/K^+^-ATPase, while antibodies raised against the purified Na^+^/K^+^-ATPase inhibit this enzyme without effect on the Na^+^-ATPase (Fig. 4).

## Na^+^-ATPase forms a phosphorylated intermediate

The Na^+^-ATPase can be classified among the P-type ATPases. Its Mg^2+^ dependence, sensitivity to vanadate and capacity to form a phosphorylated intermediate from ATP or Pi are the strongest pieces of evidence for this classification. It can be phosphorylated from inorganic phosphate in an ion-sensitive reaction stabilized by furosemide [164]. In that article, a phosphorylated polypeptide of about 100 kDa was identified for the first time as directly associated with the Na^+^-ATPase. In 2005, del Castillo et al. [42] reported a phosphorylated intermediate obtained from [^32^P]-ATP associated with the purified Na^+^-ATPase. The phosphorylation was Mg^2+^ dependent, vanadate-sensitive and stimulated by Na^+^ with two different *K*_m_ values (0.66 and 15 mM). The stimulatory effect was specific for Na^+^ and independent of anions. Phosphorylation was insensitive to ouabain but stimulated by furosemide with an EC_50_ of 1.8 ± 0.54 mM. In addition, 0.5 mM ADP partially (50 %) inhibited it. Phosphorylation was also sensitive to alkaline pH and hydroxylamine, suggesting an acyl-phosphate bond associated with the 100 kDa polypeptide of the enzyme.

A minimum reaction cycle for the Na^+^-ATPase was proposed in which the enzyme has an E1 form that can be phosphorylated from ATP in the presence of Mg^2+^ and Na^+^, producing the E1.P.Na form, sensitive to ADP. Furosemide stabilizes the E1.P.Na form. The enzyme then changes to the E2.P.Na form, insensitive to ADP, which is susceptible to dephosphorylation. A conformational change induces Na^+^ translocation through the membrane. Later, a phosphorylated intermediate associated with the ouabain-insensitive Na^+^-ATPase was identified by De Souza et al. [39] in microsomal fractions of cultured MDCK I cells and by Ventrella et al. 2010 [172] in homogenate fractions of rat kidney and microsomal fractions of rainbow trout gills. Both articles have several discrepancies, but the most important is that furosemide totally inhibits the Na^+^-stimulated phosphorylation in MDCK cells but enhances phosphorylation in rat kidney and trout gills. The data emerging from these studies, which used homogenates or microsomal fractions in which different ATPase and phosphatase activities coexist, are very difficult to interpret. However, the results obtained with the purified Na^+^-ATPase demonstrated that furosemide stabilizes the phosphorylated intermediate in an E1.P.Na form, sensitive to ADP, increasing the observed phosphorylation [45].

## Cloning of the ouabain-insensitive Na^+^-ATPase (*atna* cDNA)

The *atna* complementary DNA (cDNA) that codes for the ouabain-insensitive, K^+^-independent, Na^+^-ATPase was recently cloned from guinea pig intestinal epithelial cells (EF489487.2, GI:283442232) [140]. It was amplified by two strategies based on degenerate PCR.

The first approach was based on the use of degenerate primers designed from consensus sequences for the two best-conserved P-type ATPase structural motifs (the phosphorylation and nucleotide-binding motifs), since the ouabain-insensitive Na^+^-ATPase has features of this protein family. This strategy allowed seven P-type ATPase cDNAs to be cloned, which belonged to subtypes P_2A_ (SERCA2 and SERCA3), P_2B_ (PMCA1 and PMCA4), and P_2C_ (AT12A, AT1A1 and ATNA) [141]. They included a new ATPase cDNA fragment of 902 bp, strongly related to *atp1a1*, which was named *atna*.

The second strategy was based on successive reverse transcription PCR (RT-PCR) and hemi-nested PCR, which employed primers targeted to the three peptides identified by tandem-mass spectrometry of the purified ouabain-insensitive Na^+^-ATPase [140]. Interestingly, these three peptides are shared by the α-subunit of the Na^+^- and Na^+^/K^+^-ATPases. As expected, when this strategy was applied, two different cDNA fragments were cloned: one fragment corresponded to the α1-isoform of Na^+^/K^+^-ATPase (AT1A1) and the other matched with the *atna* fragment, cloned in the first strategy.

The sequence of guinea pig *atna* cDNA was completed by RLM-RACE for 5′- and 3′ ends. It has 2,787 nucleotides that include the following: (a) the 5′-untranslated region (5′-UTR) of 163 residues that begins with adenosine; (b) an open reading frame (ORF) of 2,436 bases that encodes a protein with 811 amino acids; and (c) a 3′-untranslated region (3′-UTR) 188 bases long in which the polyA-signal and polyA-site, necessary for messenger RNA (mRNA) maturation, were identified [140]. It was demonstrated that this cDNA codes for the ouabain-insensitive Na^+^-ATPase through silencing experiments in MDCK cells, a dog kidney cellular lineage that express a K^+^-independent, ouabain-insensitive Na^+^-ATPase [39, 140]. The *atna* cDNA was cloned from MDCK cells, employing the second strategy applied in guinea pig. A specific small-interfering RNA was designed from this cDNA sequence, and interference experiments were performed in MDCK cells. The silencing of the *atna* cDNA specifically inhibited both the ouabain-insensitive Na^+^-ATPase activity and the expression of its α-subunit [140].

## Structural analysis of ATNA protein

**Fig. 5:**
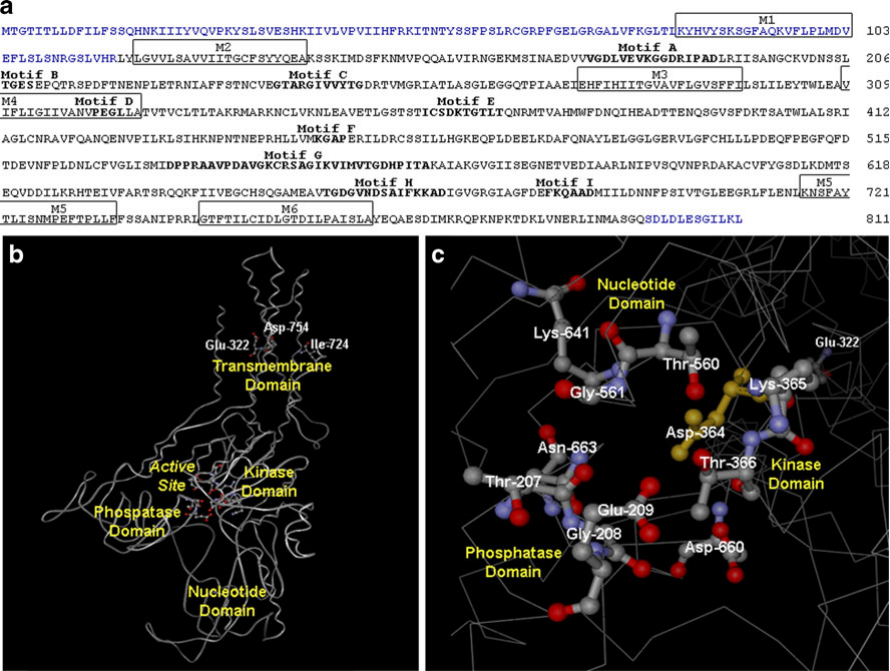
Structural features of ATNA protein. **a** Analysis of ATNA amino acid sequence. Primary structure of guinea pig ATNA (ATNA_CAVPO: EF489487.2) consisting of 811 amino acid residues is shown. Transmembrane segments (M1–M6), predicted by ClustalW [80] alignment homology and analyzed through TMpred and MPEx [160], are enclosed in *squares*. P-type ATPase structural motifs are emphasized in *bold*. The protein segment included in the 3D-prediction analysis (shown below in **b**) black letters. **b** Predicted 3D-structure of ATNA. Three-dimensional structure of ATNA_CAVPO was iteratively modeled with the non-redundant PDB database by CPHmodels 3.0 [109]. This program aligned the query amino acid sequences with a segment of the rat Na^+^/K^+^-ATPase (AT1A1_RAT: P06685), employed as template, whose partial crystalline structure had been previously determined in the E2-P conformation [69]. A lateral view of the protein is shown, showing P-type ATPase structural domains, active site and some relevant amino acid residues (Glu-322, Ile-724, and Asp-754). Only the relevant residues are shown in *ball and stick representation*. The amino acids Glu-322 and Asp-754 conform to the cation-binding sites I and II. **c** 3D structure of ATNA active site. A close-up of the ATNA_CAVPO active site is shown from the cytosolic perspective employing the three-dimensional structure obtained in **b**. Only the relevant amino acid residues are shown in ball and stick representation. The phosphorylatable Asp-364 is highlighted in *yellow*. The conformation of the nucleotide binding (Thr-560, Gly-561, Lys-641, Asp-660, and Asn-661), kinase (Asp-364, Lys-365, and Thr-366) and phosphatase (Thr-207, Gly-208, and Glu-209) domains are indicated. The cation-binding site I (Glu-322) is also shown

The ATNA-encoded protein has 811 amino acids with a probable molecular weight of 88,940 Da and an estimated p*I* of 5.70. As shown in Fig. 5a, the amino acid sequence of the ATNA protein has all P-type ATPases structural motifs described for this protein family [76, 101, 114], including the P-type ATPase signature-motif “DKTGT(L/I)T,” the dehalogenase-motif (GDGVND) and the phosphatase-motif (“TGE”).

**Fig. 6:**
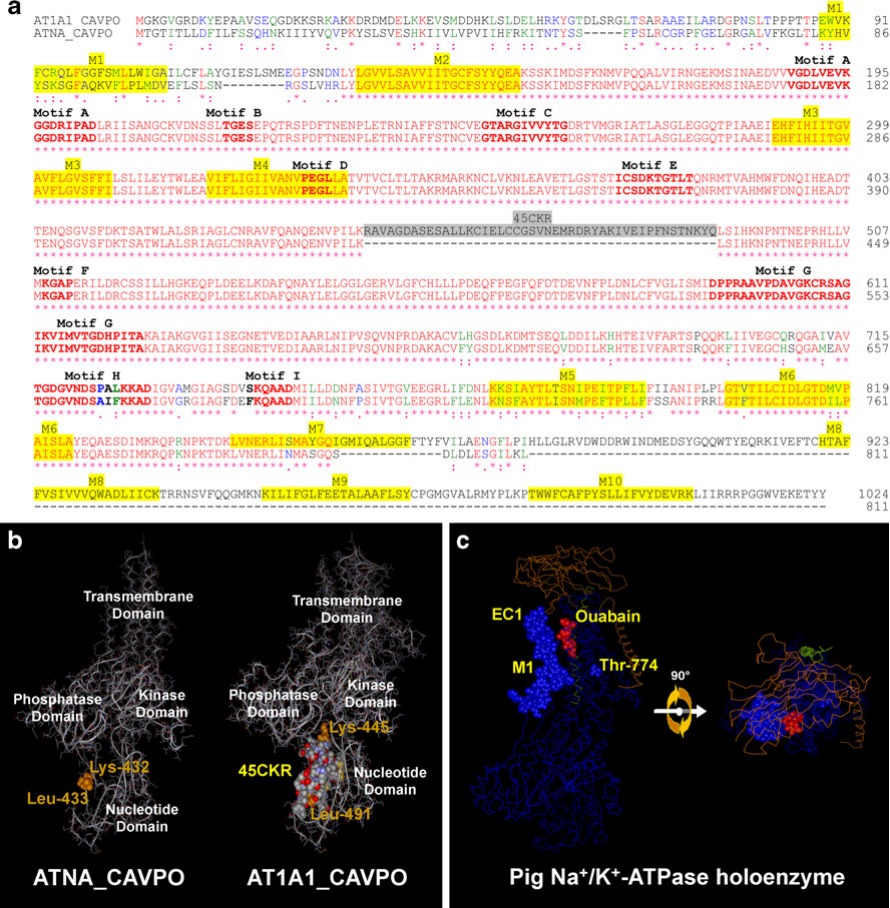
Comparative analysis of ATNA and AT1A1. **a** Alignment of ATNA and AT1A1 amino acid sequences. The amino acid sequences of ATNA_CAVPO (EF489487.2) and AT1A1_CAVPO (EF489488.2) were aligned through ClustalW 2.1 [80] with the default setting. The classic alignment style is shown: identical residues in *red* (*asterisk*), high-similarity residues in *green* (*colon*), low-similarity residues in *blue* (*period*), and different residues in *black*. P-type ATPase structural motifs are emphasized in *bold*. Transmembrane segments, predicted by homology, are highlighted with *yellow shadow*. The region (45CKR) of 45 amino acids, present in all cation/K^+^-ATPases but absent in the ATNA protein, is emphasized with gray shadow. **b** Three-dimensional comparison between ATNA and AT1A1 from guinea pig. Predicted 3D structures of ATNA_CAVPO and AT1A1_CAVPO were iteratively modeled with the non-redundant PDB database by CPHmodels 3.0 [109] (as described in Fig. 5b). Lateral views of both proteins are shown, revealing P-type ATPase structural domains. The atoms of some relevant amino acid residues are represented as space fill. The location of 45CKR in the AT1A1_CAVPO structure is indicated with standard chemical element colors. The neighbor amino acid pair delimiting the 45CKR in AT1A1_CAVPO (Lys-445 and Leu-491) and the homologous amino acid residues of ATNA_CAVPO are marked in orange. **c** Chemical interaction between AT1A1 and its inhibitor ouabain. The crystallized 3D structure of the Na^+^/K^+^-ATPase holoenzyme from pig is shown in E2-P conformation with ouabain-bound (PDB: 3N23). The Na^+^/K^+^-ATPase alpha, beta and gamma chains are colored *blue*, *orange*, and *green*, respectively. Ouabain is marked in *red*. The atoms of ouabain and the amino acid residues in the M1 and EC1 segments of AT1A1_CAVPO are represented as space fill. Two views are shown: lateral (*left*) and extracellular (*right*) after 90° lateral rotation

The amino acid residues considered essential for P-type ATPase function [15, 103] seem to be present in ATNA. Sequence alignment through ClustalW [80] (Fig. 6a) and three-dimensional topology prediction by CPHmodels 3.0 program [109] (Fig. 5b, c) allow the homologous residues at the corresponding positions described for AT1A1_PIG and SERCA1_RABIT ATPases, whose crystalline structure was previously elucidated [112, 157, 169], to be identified in ATNA. The homology comparison is summarized in Table 1. In fact, all essential residues are identical in ATNA and AT1A1 and differ in only one position from SERCA1 (Asp-754 changes to Asn-796 in SERCA1). Although it is reasonable to suppose that homologous residues play similar functions, this requires experimental demonstration. Nevertheless, homology analysis strongly suggests that ATNA_CAVPO has the amino acid residues essential for ATP hydrolysis (Fig. 5c) [15, 103], including the phosphorylatable amino acid (Asp-364) and the residues necessary for nucleotide binding (Thr-560, Gly-561, Lys-641, Asp-660, and Asn-663), enzyme phosphorylation (Asp-364, Lys-365, and Thr-366 from the kinase domain) and enzyme dephosphorylation (residues Thr-207, Gly-208, and Glu-209 from the phosphatase domain).

**Table 1:** Highly preserved amino acid residues implicated in P-type ATPase function

ATNA_CAVPO residue	ATP1A1_CAVPO homolog residue	ATP1A1_PIG homolog residue	SERCA1_RABIT homolog residue	Location	Function
Thr-207	Thr-220	Thr-212	Thr-181	Motif B, phosphatase domain	Enzyme dephosphorylation
Gly-208	Gly-221	Gly-213	Gly-182	Motif B, phosphatase domain	Enzyme dephosphorylation
Glu-209	Glu-222	Glu-214	Glu-183	Motif B, phosphatase domain	Enzyme dephosphorylation
Glu-322	Glu-335	Glu-327	Glu-309	Motif D, M4, transmembrane domain	Cation-binding sites I and II
Asp-364	Asp-377	Asp-369	Asp-351	Motif E, kinase domain	Phosphorylable residue
Lys-365	Lys-378	Lys-370	Lys-352	Motif E, kinase domain	Enzyme phosphorylation
Thr-366	Thr-379	Thr-371	Thr-353	Motif E, kinase domain	Enzyme phosphorylation
Thr-560	Thr-618	Thr-610	Thr-625	Motif G, nucleotide-binding domain	Nucleotide binding
Gly-561	Gly-619	Gly-611	Gly-626	Motif G, nucleotide-binding domain	Nucleotide binding
Lys-641	Lys-699	Lys-691	Lys-684	Nucleotide-binding domain	Nucleotide binding
Asp-660	Asp-718	Asp-710	Asp-703	Motif H, nucleotide-binding domain	Nucleotide binding
Asn-663	Asp-721	Asp-713	Asn-706	Motif H, nucleotide-binding domain	Nucleotide binding
Ser-725	Ser-783	Ser-775	Ser-767	M5, transmembrane domain	Cation binding
Asn-726	Asn-784	Asn-776	Asn-768	M5, transmembrane domain	Cation binding
Glu-729	Glu-787	Glu-779	Glu-771	M5, transmembrane domain	Cation binding
Asp-754	Asp-812	Asp-804	Asn-796	M6, transmembrane domain	Cation-binding site II
Asp-758	Asp-816	Asp-808	Asp-800	M6, transmembrane domain	Cation-binding site II

Essential amino acid residues of ATNA_CAVPO (EF489487.2) and its homologues in AT1A1_CAVPO (EF489488.2), AT1A1_PIG (P05024), and SERCA1_RABIT (P04191) are shown. Domain location and function are also indicated

Additionally, TMpred and MPEx [160] programs predict at least six transmembrane α-helices (M1–M6) that match the P-type ATPase core-protein, which is considered to be constituted by four characteristic domains: nucleotide binding, kinase, phosphatase, and transmembrane [90]. In this sense, the segments M1, M2, M4, M5, and M6 seem to form the half-channels for Na^+^ transport. Moreover, ATNA seems to have the essential amino acid residues for cation transport, following a model of alternating access [103] without counter-ions. The relevant residues for this model include Glut-322, Ser-725, Asn-726, Glu-729, Asp-754, and Asp758. The residues Glut-322 and Asp-754, respectively, located in M4 and M6, seem to be involved in Na^+^ binding. Thus, Glu-322 would constitute the Na^+^-binding site I, while site II should be simultaneously formed by Glu-322 plus Asp-804 [4, 15, 32, 103]. Additional residues such as Ser-725, Asn-726, Glu-729, and Asp758 may also participate in cation coordination. The segment M1 of ATNA has the residue Leu-98, which would function as the cation gate-lock for Na^+^ occlusion after enzyme phosphorylation and during the E1P-E2P transition [15, 103]. Therefore, ATNA could pump one or two Na^+^ ions per catalytic cycle. These analyses suggest that ATNA has the necessary elements to couple the exergonic hydrolysis of ATP with the endergonic transport of Na^+^ against its electrochemical gradient.

## Comparison between Na^+^- and Na^+^/K^+^-ATPases (ATNA and AT1A1 proteins)

ATNA protein has 64 % identity and 72 % high similarity to AT1A1 protein from guinea pig [140]. However, the differences are not uniformly distributed along their primary structures but grouped as clusters at the amino and carboxyl terminal ends (Fig. 6a). In addition, ATNA lacks a region of 45 amino acids (45CKR) present in the nucleotide-binding domain of all cation/K^+^-ATPases, including AT1A1 [140, 141].

Some features could explain the functional differences observed between the K^+^-independent, ouabain-insensitive Na^+^-ATPase (ATNA) and the Na^+^/K^+^-ATPase (AT1A1). The three-dimensional structure prediction using CPHmodels 3.0 [109] shows that 45CKR is located between the phosphatase domain and the phosphorylation site in the E2P conformation. In the cation/K^+^-ATPases, we have proposed [140] that the 45CKR could prevent the approximation of the phosphatase domain to the phosphoryl-aspartate, stabilizing the phosphoryl-enzyme in its E2P conformation until K^+^ is bound. Once K^+^ is bound, it should induce an additional conformational change that permits the phosphatase domain to interact with the aspartyl-phosphate and the subsequent dephosphorylation of the enzyme. The absence of 45CKR in ATNA could permit direct dephosphorylation, without K^+^ binding, which would explain the K^+^-independence of ATNA.

Two structural characteristic of ATNA in M1-EC1 and M5 could explain its ouabain resistance. The segments M1 and EC1 of the Na^+^/K^+^-ATPase α-subunit have been implicated in ouabain binding [112, 157]. Figure 6c shows the pig Na^+^/K^+^-ATPase holoenzyme bound to ouabain (red space-filling atoms), which interacts closely with the M1 and EC1 segments of the enzyme (blue space-filling atoms). These segments show important modifications in the amino acid sequence of ATNA (Fig. 6a) that could preclude ouabain binding. Moreover, the residue Thr-774, present in M5 of AT1A1 (Fig. 6c), is replaced by Ile-724 in ATNA_CAVPO (Fig. 6a). It has been shown that the single substitution of Thr-774 by alanine transforms the Na^+^/K^+^-ATPase in an ouabain-insensitive enzyme [70]. Thus, the replacement of this threonine residue by isoleucine (Ile-724) in ATNA could result in the characteristic ouabain-insensitivity of this enzyme.

## Phylogenetic analysis of ATNA

**Fig. 7:**
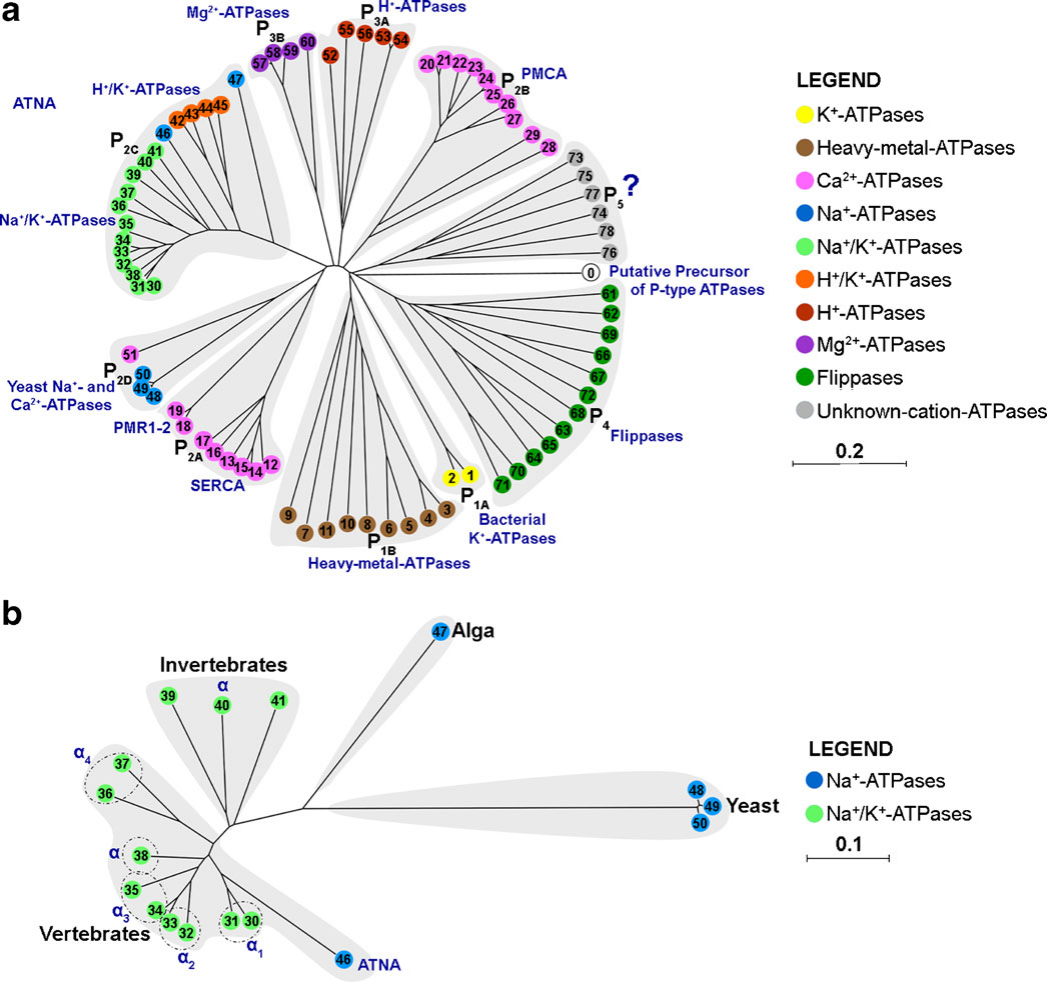
Phylogenetic analysis of ATNA. **a** Phylogenetic tree of P-type ATPase family. A total of 78 amino acid sequences of P-type ATPases, representing the ten sub-types described (P1A-P5), as indicated, were aligned through ClustalW 2.1 [80]. The transported cations are indicated with colors: K^+^ (*yellow*), heavy metals (*brown*), Ca^2+^ (*hot pink*), Na^+^ (*blue*), Na^+^ and K^+^ (*light green*), H^+^ and K^+^ (*salmon*), H^+^ (*orange*), Mg^2+^ (*purple*), amino-phospholipids (*dark green*), and unknown cations (*gray*). The clue numbers and their respective Swiss-prot access numbers of the aligned sequences are 1:P03960, 2:Q8Z8E5, 3:Q04656, 4:P70705, 5:Q64446, 6:Q9XT50, 7:Q9M3H5, 8:Q9S7J8, 9:Q59465, 10:O08462, 11:Q59207; 12:P13585, 13:P70083, 14:Q03669, 15:Q00779, 16:Q9YGL9, 17:Q93084, 18:P57709, 19:Q80XR2, 20:Q00804, 21:P11505, 22:Q9R0K7, 23:P58165, 24:Q16720, 25:Q64568, 26:P23634, 27:Q64542, 28:P38929, 29:Q37145, 30:P09572, 31:(GI:EF489488.2), 32:P50993, 33:P06686, 34:P13637, 35:P58312, 36:Q13733, 37:Q64541, 38:P05025, 39:P28774, 40:P13607, 41:P35317, 42:P50996, 43:Q92126, 44:Q92036, 45:Q64392, 46:(GI: EF489487.2), 47:Q9SXK5, 48:P13587, 49:Q01896, 50:Q12691, 51:P22189, 52:Q58623, 53:P11718, 54:P12522, 55:P20649, 56:P09627, 57:P0ABB8, 58:P0ABB9, 59:P36640, 60:P22036, 61:O54827, 62:O94823, 63:P98197, 64:Q9N0Z4, 65:Q8NB49, 66:Q29449, 67:P98200, 68:O43520, 69:P98204, 70:O70228, 71:O43861, 72:P39524, 73:Q9CTG6, 74:Q9HD20, 75:Q9H7F0, 76:O14072, 77:O74431, and 78:Q9LT02. The putative P-type ATPase phylogenetic precursor (Q58378) from *Methanococcus jannaschii* was included in the alignment (*white circle*, clue number: 0). Unrooted dendrograms were drawn with Unrooted.exe using the ClustalW alignment data. Defined phylogenetic branches are shadowed in *gray* and ATPase sub-types are indicated. **b** Phylogenetic tree of sodium-stimulated P-type ATPases. ClustalW aligment and Unrooted dendrogram drawn was repeated with only sodium-stimulated P-type ATPases (clue numbers 30–41 and 46–50). Defined phylogenetic branches are shadowed in *gray* and taxonomic lineages are indicated. The transported cations are indicated with colors: Na^+^ (*blue*) or Na^+^ plus K^+^ (*light green*). *PMCA* plasma membrane Ca^2+^-ATPases, *SERCA* sarcoplasmic/endoplasmic reticulum Ca^2+^-ATPases, *PMR1-2* Golgi apparatus membrane Ca^2+^-ATPases, *ATNA* guinea pig ouabain-insensitive Na^+^-ATPase. The α–isoforms of the Na^+^/K^+^-ATPase are indicated

Sequence alignment of 78 P-type ATPases of all sub-types described was performed by ClustalW 2.1 [2] and the un-rooted dendrogram was drawn with Unrooted.exe. The resulting phylogenetic tree is shown in Fig. 7a. As expected, ten clearly defined branches were identified, corresponding to the ten described sub-types. These results situate ATNA in sub-type IIC (P_2c_), which includes the four α-isoforms of the Na^+^/K^+^-ATPase (AT1A1-4), the two α-isoforms of the H^+^/K^+^-ATPase (ATP4A and ATP12A), the invertebrate α-subunit of Na^+^/K^+^-ATPase, and the Na^+^-ATPase from the alga *Heterosigma akashiwo*.

The sequence alignment of only Na^+^-stimulated ATPases identified four branches, each corresponding to a single taxonomic lineage: yeast, alga, invertebrate, and vertebrate (Fig. 7b). As expected, the vertebrate and invertebrate ATPases are more closely phylogenetically related. For example, the Na^+^/K^+^-ATPases from the vertebrate *Torpedo californica* (SP:P05025, clue number 38), and the invertebrate *Artemia sanfranciscana* (SP:P28774, clue number 39) are 77 % identical. In contrast, the artemia ATPase is only 26 % identical to the Na^+^-ATPase from *Saccharomyces cerevisiae* (SP:Q01896, clue number 50). This suggests that Na^+^-stimulated ATPases from vertebrates and invertebrates have diverged recently, conforming with close phylogenetic groups in the animal kingdom, and their Na^+^ dependence seems to have originated independently of similar Na^+^-ATPases from plants and fungi.

ATNA shares the highest identity with AT1A1 (64 %) in guinea pig. Homology with other Na^+^-ATPases, with unknown or doubtful K^+^ dependence, is lower. Thus, ATNA is 56 % identical to the Na^+^-ATPase from the alga *H. akashiwo* (SP:Q9SXK5, clue number 47) and 24–43 % identical to some yeast Na^+^-ATPases (SP:P13587, Q01896, and Q12691, clue numbers 48–50, respectively). These results agree with the evidence that *atna* and *atp1a1* share the same locus (*atp1a1*) and did not follow independent evolutionary pathways since they are still inseparable genes. In fact, both transcripts share exons.

The origin of *atna* seems to precede the divergence of mammals from the rest of the vertebrates since it is broadly spread in this taxonomic class. The *atna* gene could have been generated from insertional events plus the duplication of some *atp1a1* exons, which followed divergent evolution.

## The ouabain-insensitive Na^+^-ATPase (*atna*) gene in guinea pig and human

**Fig. 8:**
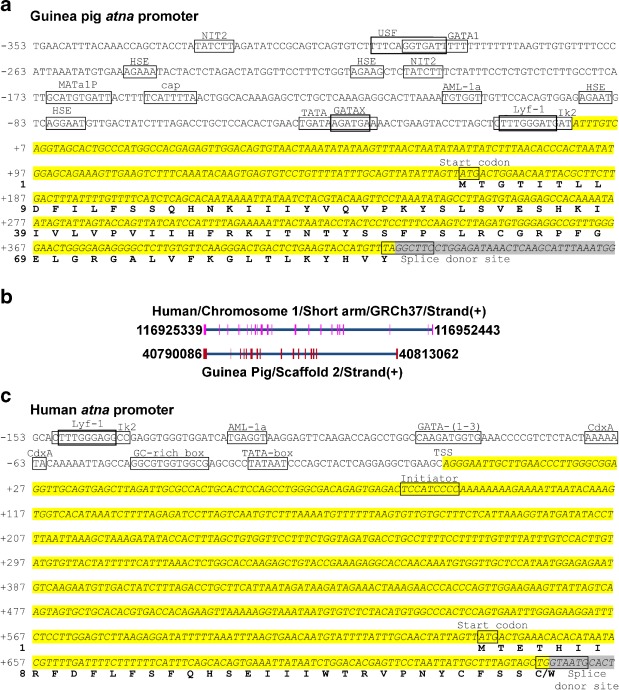
Search for *atna* gene in human and guinea pig. **a** Location of *atna* gene in genomic DNA of human and guinea pig. The human gene was identified by alignment of the amino acid sequence of ATNA_CAVPO (EF489487.2) with the human genomic DNA around locus *atp1a1* (chromosome 1, short arm, GRCh37, plus strand), translated in the three frames for both strands, employing NCBI Blast5 bl2seq TBLASTN [2]. Exon–intron distribution was defined through NCBI Spidey by alignment between the 2787-bp guinea pig *atna* mRNA (EF489487.2) and the respective genomic DNA segment for human or guinea pig. For human (*top*), Homo sapiens *atp1a1* locus from base +116925339 to base +116952443 was aligned. For guinea pig (*bottom*), *Cavia porcellus atp1a1* locus from base +40790086 to base +40813062 (scaffold 2, plus strand) was used. It allowed all exons and introns to be identified, including the ending exons. Analysis of results is shown as a proportional uni-dimensional scheme of exon/intron distribution, where exons (*pink or red boxes*) and introns (*blue connector line*) are represented at scaled width. Identification of *atna* promoters for human (**b**) and guinea pig (**c**). Once the first exon was identified, promoters were identified through BDGP Neural Network Promoter Prediction [134]. Response elements for transcriptional factors were predicted by TFSearch [67]. Transcriptional start site (TSS) is defined on +1. Promoters, initiators, transcriptional factor elements, start codons and splice donor sites are identified, enclosed in *squares*. The predicted transcribed segment is shown *shadowed*. First exon and first intron are shadowed *yellow* and *gray*, respectively. Encoded amino acid sequence for first exon is shown below in *bold script*

The search for the *atna* gene in the guinea pig genomic database reveals that *atna* and the Na^+^/K^+^-ATPase α1-isoform (*atp1a1*) cDNAs are at the same genetic locus: *atp1a1*. The *atna* mRNA shares 13 exons with *atp1a1* mRNA, but has five exclusive exons located at the 5′ and 3′ ends. The transcription start sites (TSS) for both transcripts are separated by more than 8.4 kb, suggesting that these mRNAs are independently transcribed from independent promoters. The programs Promoter Scan and TFsearch predict one putative *atna* promoter downstream of the *atp1a1* promoter. The guinea pig *atna* promoter (Fig. 8a) includes the following: (a) TATA-box, (b) two overlapping initiators (Lyf-1 and Ik2), and (c) four HSF sites [140]. These features are likely to be enough to allow the *atna* gene to be independently transcribed, as described for other genes [13, 16].

Given that the ouabain-insensitive Na^+^-ATPase has been described in other species, as indicated above, we decided to explore the existence of a putative orthologous gene in humans. Therefore, the human genome from the ENSEMBL database was analyzed with TBLASTN [2], using the ATNA amino acid sequence as input (Fig. 8b, top). The *atna* gene seems to be located in the plus strand of the locus *atp1a1*, in the short arm of chromosome 1, near to the centromere (locus GRCh37 from +116915290 to +116952883). The start codon appears to be encoded by the human genomic nucleotides +116925339 to +116925341.

The exon–intron distributions for guinea pig and human were determined using the program NCBI Spidey by aligning the 2,787-bp guinea pig *atna* mRNA (EF489487.2) and the respective genomic DNA segment. All exons of the *atna* ortholog from human were identified. The exon–intron arrangements for *atna* in the *atp1a1* loci from human (top) and guinea pig (bottom) are shown in Fig. 8b. The human *atna* seems to have 21 or 22 exons (one exon is very short) located in the *atp1a1* locus from base +116925339 to +116952443, showing an exon–intron pattern similar to the guinea pig gene.

Once the human first exon was located, the promoter was identified using the program BDGP Neural Network Promoter Prediction [134], and Response Elements for Transcriptional factors were predicted by TFSearch [67]. The TSS, represented by an adenosine residue, corresponds to base +116924704 (chromosome 1), located 164 bases upstream of the putative start codon (Fig. 8c). This predicted promoter, as in the guinea pig ortholog (Fig. 8a), includes a TATA-box, a GC-rich box and two initiators Lyf-1 and Ik2. These initiators have been involved in immune cell differentiation [71] and the inflammatory response, as negative regulators of iNOS [31] and upregulators of IL10 expression [171]. In addition, the human promoter region has another putative initiator element, 87 bases downstream of the described TSS [34, 159].

Heat-shock-factor elements (HSE) are genetic sequences located in promoter regions, recognized by heat-shock transcriptional factors (HSF), regulatory proteins that modulate gene expression [3]. It is noteworthy that the four putative HSE sites present in the *atna* promoter (from −249 to −76) are absent in the *atp1a1* gene. This opens the possibility that the expression of these two genes could be differentially regulated in response to physiological or stress situations (e.g., hormone stimulation, heat shock, nutritional starvation, and osmotic stress). HSF-1 is activated by osmotic stress, inducing several genes [20]. If HSE in the *atna* gene responds to HSF1, its overexpression would allow the cell to extract osmotic particles such as Na^+^ ions to compensate the osmotic disturbance. It is interesting that other inducers of HSF-1, such as ethanol, cell volume alteration, oxidative stress, and nutritional stress, modulate the Na^+^-ATPase activity, as mentioned above.

The presence of predicted response elements for HSF, Lyf-1, and Ik2 in the *atna* promoter allows us to hypothesize that *atna* could participate in the epithelial inflammatory response. It has been shown that HSF-1, activated in febrile states, can also modify the expression of non-HSP genes including those for cytokines and chemokines [33, 107]. Moreover, Tanaka and Mizushima [162] showed that the activation of HSF-1 protects against both irritant-induced gastric lesions and IBD-related colitis, promoting tissue repair.

## Why do cells express the K^+^-independent Na^+^-ATPase and the Na^+^/K^+^-ATPase with apparent overlapping functions in active sodium extrusion?

The identification of the *atna* gene and its encoded protein, the K^+^-independent, ouabain-insensitive Na^+^-ATPase, represents a breakthrough in the understanding of epithelial Na^+^ transport. It provides exceptional biochemical and molecular evidence to explain the multiple functional data that suggested the existence of an active Na^+^ transport, independent of the Na^+^/K^+^ pump, in renal and intestinal epithelia. The presence of the second sodium pump in the basolateral plasma membrane would allow the epithelial cells to extrude Na^+^, Cl^−^, and water under circumstances where transepithelial Na^+^ transport is highly stimulated (i.e., transepithelial Na transport coupled to sugars and amino acids), with no relevant effect on the activity of the Na^+^-K^+^ exchange pump. Under these conditions, the electroneutral movement of Na^+^ and Cl^−^ by the second sodium pump would eliminate the obligatory regulation of cell potassium concentration to maintain the membrane potential. In addition, the extrusion of Na^+^ and Cl^−^ across the basolateral membrane followed by water would permit the regulation of cell volume and water absorption without significant participation by the Na^+^/K^+^ pump. The second sodium pump could also play a similar role in non-epithelial cells, where its contribution to cell volume regulation would be predominant under isotonic conditions.

Finally, it is interesting to note that the expression of the renal and intestinal K^+^-independent, ouabain-insensitive Na^+^-ATPase is upregulated by Ang II and is increased in the kidneys of spontaneously hypertensive rats, without modification of the expression of the Na^+^/K^+^-ATPase. These observations suggest that the Na^+^-ATPase, as an essential participant in sodium absorption, could determine the development of salt-dependent essential hypertension. Furthermore, the recognition of particular regulatory sites in its promoter region, different from those identified in the Na^+^/K^+^-ATPase gene, opens the possibility that the two enzymes could be differentially regulated under some physiological (e.g., osmotic and volume regulation, hormonal response) or pathophysiological (e.g., essential hypertension, inflammation) conditions.

## Future perspectives

The purification and characterization of the Na^+^-ATPase raises several questions that need to be elucidated. The identification of a putative β-subunit in the purified enzyme, which has not yet been cloned, opens the question whether this subunit is essential for enzyme function or is an insertion chaperone. The answer will probably come from expression experiments. Additionally, the expression of the α or α/β holoenzyme in heterologous systems will allow enough recombinant enzyme to be produced for NMR and crystallization experiments, whereby the functional structure of this protein will be determined. In addition, the recombinant enzyme will permit the exploration of site-directed mutations and thus the identification of essential residues and structural domains. Furthermore, recognition of the inhibitory site for furosemide or triflocin through structural and biochemical studies will allow us to design inhibitory molecules with potential clinical use (e.g. as diuretics). The predictions obtained by in silico analysis will be the starting points for new experimental approaches to elucidate and/or to confirm the biochemical and physiological characteristics of the Na^+^-ATPase. For instance, the identification of multiple regulatory elements in its promoter region forces detailed molecular analysis of this region and comparison with that of the Na^+^/K^+^-ATPase in terms of Na^+^ transport regulation. The definitive demonstration of the role of Na^+^-ATPase in pathological states such as inflammatory diseases or essential hypertension will undoubtedly exert a significant impact on medicine.
